# Language Development in the Second Year of Life: The Case of Children with Sex Chromosome Trisomies Diagnosed before Birth

**DOI:** 10.3390/ijerph19031831

**Published:** 2022-02-06

**Authors:** Laura Zampini, Alessandra Lorini, Gaia Silibello, Paola Zanchi, Francesca Dall’Ara, Paola Francesca Ajmone, Federico Monti, Faustina Lalatta, Maria Antonella Costantino, Paola Giovanna Vizziello

**Affiliations:** 1 Department of Psychology, University of Milano-Bicocca, Piazza dell’Ateneo Nuovo 1, 20126 Milan, Italy; a.lorini5@campus.unimib.it (A.L.); paola.zanchi@unimib.it (P.Z.); 2 Child and Adolescent Neuropsychiatric Unit, Foundation IRCCS Ca’ Granda Ospedale Maggiore Policlinico, via Pace, 9, 20122 Milan, Italy; gaia.silibello@gmail.com (G.S.); francesca.dallara1@gmail.com (F.D.); paola.ajmone@policlinico.mi.it (P.F.A.); monti.federico80@gmail.com (F.M.); antonella.costantino@policlinico.mi.it (M.A.C.); paola.vizziello@policlinico.mi.it (P.G.V.); 3 Clinical Genetics Unit, Foundation IRCCS Ca’ Granda Ospedale Maggiore Policlinico, via della Commenda, 12, 20122 Milan, Italy; faustina.lalatta@fastwebnet.it

**Keywords:** predictive indices, sex chromosome trisomies, longitudinal, language outcomes, late-talking

## Abstract

Many individual factors, such as early communicative skills, could play a role in explaining later linguistic outcomes. The detection of predictive variables is fundamental to identifying early the children who need intervention. The present study focuses on children with sex chromosome trisomies (SCTs), genetic conditions with an increased risk of developing language delays or impairments. The aims are to analyse their communicative skills at 18 months of age, and identify significant predictors of their later vocabulary size. Participants were 76 18-month-old children (38 with SCTs, and 38 typically-developing (TD) children). Their communicative skills were assessed during a parent–child play session, and parents filled in a report on their vocabulary development at 18 and 24 months. Children with SCTs showed significantly poorer linguistic skills at 18 months in both preverbal (babbling and gestures) and verbal abilities. A high percentage (nearly 70%) of toddlers with SCTs were late-talking children at 24 months, and those toddlers showed a lower frequency of babbling utterances at 18 months. Early lexical skills, children’s developmental quotient, and being part of the group of toddlers with SCTs were significant predictors of children’s vocabulary size six months later. These variables should be considered when assessing the linguistic competence of a child with SCTs to detect possible early risk factors of future language impairment.

## 1. Introduction

Early detection of children with delayed or impaired language development is fundamental, considering the potential impact of precocious linguistic difficulties on later development [1]. However, identifying children at risk for a developmental language disorder (DLD) is not an easy task, owing to the high individual variability that characterises the first stages of communicative development [2,3].

The hypothesis of continuity in language development states the existence of stability of individual differences across the preverbal to the verbal stage, and across the period from first words to grammar [4,5,6]. In this light, finding a relationship between preverbal measures of communicative skills and later vocabulary development could be useful to identify those variables that can predict children’s language status at a later stage. Many studies analysed children’s preverbal production to identify the best predictors of later vocabulary skills [7]. One of the most studied and validated measures is babbling production: both the onset of canonical babbling [8,9] and babbling structure [10] were identified as significant predictors of later vocabulary size in typically-developing (TD) children. Even gesture production has been identified as a predictive index of later lexical skills [5,11]. In particular, pointing gesture emerged as a key behaviour in language development [5,12].

Besides early preverbal communicative skills, motor development [13], general-domain cognitive abilities [14], and first-word production [15,16] are other individual variables connected to later language development. However, linguistic outcomes can be influenced not only by individual factors but also by various environmental and genetic factors. Regarding environmental aspects, socio-economic status and maternal education [17,18] are frequently detected as predictors of later lexical development. Concerning the genetic factors, a significant role in predicting children’s linguistic outcomes is played by family history of language impairments [19] or by the presence of genetic anomalies, for instance, having a genetic syndrome characterised by a neuropsychological profile with a particular disadvantage in verbal skills (e.g., 22q11.2 deletion syndrome [20]).

The present study focuses on a specific population, children with sex chromosome trisomies (SCTs), aiming to analyse this genetic condition’s impact on children’s language development. Moreover, we aim to analyse the effects of early individual characteristics in determining children’s vocabulary development within this biological condition. We chose to focus on this population since one of its most distinctive traits is the extra chromosome’s impact on language development [21].

SCTs are genetic syndromes characterised by an extra sex chromosome to a normal karyotype. The extra chromosome could be an X, leading to triple X syndrome in females (47, XXX) and Klinefelter syndrome in males (47, XXY), or a Y, leading to Jacobs syndrome in males (47, XYY). The estimated prevalence of these conditions ranges from about 1 in 500 to 1 in 1000 same-sex individuals [22,23]. However, it should be noted that this prevalence could be underestimated, owing to their mild physical and cognitive phenotype. The neuropsychological profile of children with SCTs is frequently characterised by language and learning impairments, attentional and executive deficits, and atypical motor development [23,24,25]. The IQs usually have a normal distribution, but with the mean score of about 10 points lower than TD children, and verbal abilities at a lower level than the performance ones [26,27]. Children with SCTs show a higher incidence of language delays and impairments [27,28,29,30], and their neuropsychological profile is similar to that of children with DLD (i.e., a significant impairment in verbal skills, with general intelligence usually in the normal range, but with the mean slightly shifted to the left). No significant differences among the three SCT karyotypes were found in the first stages of language development [21,31], though some differences in their profiles could emerge later. For instance, Lee et al. [32] suggested that an additional X chromosome (as in triple X syndrome and Klinefelter syndrome) might have a greater effect on structural language abilities, whereas an additional Y (as in Jacobs syndrome) might have a higher impact on pragmatics.

Recent literature reports that a slowdown in language development could be highlighted from the first stages of acquisition in all of the SCTs [21,27]. As early as eight months of age, children with SCTs (*n* = 26) showed a delay in preverbal communicative skills. Specifically, a significantly lower number of children with SCTs than TD toddlers showed the ability to babble during a parent–child interactive play session; in addition, the frequency of production of canonical and reduplicated babbling was significantly lower in toddlers with SCTs than in the control group [27]. In the second year of life (11–23 months), children with SCTs (*n* = 35) showed poorer receptive and expressive semantic skills than TD peers, according to both parent reports and neuropsychological assessments [21]. Zampini, Draghi, et al. [30], at two years of age, identified 60% of children with SCTs (*n* = 9 out of 15 children) as late-talking toddlers (i.e., children with a vocabulary size smaller than 50 words at 24 months). Moreover, during a play session in interaction with a parent, these children produced a significantly lower proportion of verbal utterances, and a significantly higher number of pointing gestures than TD children [30]. As Urbanus et al. [21] reported in their recent cross-sectional study on 103 children ranging in age from one to six years, the linguistic performance of individuals with SCTs showed large deviations from the control group on nearly all language domains, and the number of children who experience linguistic problems increased with age. Therefore, monitoring language development from the earliest stages of nonverbal communication is fundamental to identifying children at risk, and intervening when needed [21].

To date, only a few studies investigated the possible early predictive indices of later linguistic competence in children with SCTs [27,30,33]. The infants’ communicative productions at eight months (*n* = 21) did not appear related to vocabulary skills at 24 months [27]. In contrast, the number of communicative gestures produced at 18 months by children with Klinefelter syndrome (*n* = 13) appeared significantly related to their vocabulary size six months later [33]. Moreover, early pre-syntactic abilities (i.e., transitional forms, as horizontal repetitions or formulas, and word combinations) produced by children with SCTs at 24 months (*n* = 13) appeared to be significant predictors of the linguistic outcome of the same children at four years [34].

Focussing on the population of children with SCTs, in particular on individuals identified by prenatal screening and diagnosed before birth, provides the possibility of studying the early stages of communicative development in children who have a higher probability of developing language impairments. Therefore, it allows us to investigate the emergence and trajectories of DLD, and detect early markers of language delays or impairments [21].

### Aims of the Study

The study’s first aim is to verify the impact of having a diagnosis of SCTs on language development during the second year of life. In particular, we aim to analyse the existence of significant differences in the communicative skills exhibited at 18 months by children with SCTs and TD toddlers. According to the previous literature, we expect to find lower linguistic competencies in children with SCTs. However, only a few data are available for this age, and preverbal skills (vocal and gestural) have been only marginally analysed to date.

The second aim is to verify the presence of individual variability in the early communicative development of children with SCTs, and its predictive role on later linguistic outcomes. In particular, we aim at identifying which individual factors (among preverbal vocal and gestural communicative skills, developmental quotient, and early lexical abilities) are the best predictors of vocabulary outcomes at 24 months.

## 2. Materials and Methods

### 2.1. Participants

Participants were 76 18-month-old children: 38 with SCTs (14 with XXX, 12 with XXY, and 12 with XYY) (age: M = 18 months; SD = 0.73; range = 17–21 months), and 38 TD children (15 females) (age: M = 18 months; SD = 0.49; range = 17–20 months). The children with SCTs were recruited through individual contact with families participating in a monitoring programme at the Child and Adolescent Neuropsychiatric Unit of the Foundation IRCCS Ca’ Granda Ospedale Maggiore Policlinico (Milan, Italy). The TD toddlers were selected from a sample of children involved in a research project on language development at the Department of Psychology of the University of Milano-Bicocca (Milan, Italy). The children in both groups were then assessed at 24 months. Twenty-three out of thirty-eight children with SCTs, and twenty-two out of thirty-eight TD children participated in Zampini et al.’s study (2021) when they were eight months old.

All children with SCTs were identified by prenatal screening (e.g., NIPT), and diagnosed before birth by amniocentesis or chorionic villus sampling. No foetal anomalies were detected in the children who participated in the study. Since the participants with SCTs were all identified before birth, the group was relatively unbiased compared to participants of studies based on cases identified during investigations for developmental problems. All of the children (with SCTs and TD) had normal hearing and no history of ear infections, and none of them had any additional genetic or neurological condition. Moreover, all the participants came from monolingual Italian-speaking families. Children’s parents signed a written informed consent form before inclusion in the project. No incentives were provided to the participants.

### 2.2. Procedure

The children’s communicative production was assessed by analysing their spontaneous vocal and gestural acts during a parent–child play session. Parents were instructed to play as usual with their children using three sets of toys: a farm with plastic animals, some children’s illustrated books, and a doll with a nurturing set. The sessions were video-recorded and lasted about 15 min. The examiner observed and video-recorded the sessions behind a unidirectional mirror. Then, trained observers transcribed in CHAT format [35] exactly 15 min of interaction for each child.

We used the Griffiths Mental Development Scales (GMDS) [36] to assess children’s psychomotor development, and a parental inventory to assess their vocabulary development. Specifically, parents filled out the Italian version of the MacArthur-Bates Communicative Development Inventories—Words and Gestures form (Il Primo Vocabolario del Bambino (PVB) [37]). From the administration of the GMDS, we obtained children’s Developmental Quotient (DQ), whereas from the PVB, we obtained children’s vocabulary size (i.e., the number of words that children could spontaneously use).

When children reached 24 months (M = 24; SD = 0.50), they participated in another assessment session, and their vocabulary size was assessed re-evaluated using the PVB. Owing to dropout, PVB production at 24 months was available for 62 children (30 with SCTs, and 32 TD). Vocabulary size at 18 months was not significantly different between children who participated at the 24-month follow-up and children who dropped out of the project, nor in children with SCTs (U = 79.5; *p* = 0.749) or TD children (U = 79; *p* = 0.664).

### 2.3. Measures of Communicative Production

Both vocal and gestural productions were coded. Concerning vocal production, children’s communicative acts were classified as follows:

- Vocalisations: This category includes communicative grunts (e.g., ‘mh’) and vocalisations (e.g., ‘ah’).
- Babbling: This category includes babbling (e.g., ‘ba’; ‘baba’; ‘dapapadada’) and non-words (i.e., productions with the phonotactic structure of a word, but without meaning).
- Verbal utterances: This category includes utterances composed of at least a meaningful word. To assign the status of a meaningful word, we used the criteria suggested by Vihman and McCune [38], which are an appropriate context of use and similarity to adult vocalisation shape. The verbal utterance category includes single-word utterances (e.g., ‘farfalla’ (butterfly)) and multi-word utterances (e.g., word combinations, such as ‘apro porta’ (open door)).

From the 15 min of transcription, we computed the following measures:

1. Number of utterances produced: According to D’Odorico and Jacob [39], we considered as an utterance a vocal production uttered in a unique conversational turn, separated from other productions by a pause longer than 1 s.
2. Number of vocalisations.
3. Number of babblings.
4. Number of verbal utterances.
5. Tokens (i.e., the total number of meaningful words uttered).
6. Types (i.e., the number of different words uttered).

Children’s word types were also classified in terms of word classes: nouns, routines (e.g., ‘grazie’ (thank you), ‘ciao’ (bye), and proper nouns), verbs, adjectives, and functional words. The proportion of each word class on the total number of types uttered was computed.

Concerning gesture production, the children’s communicative gestures produced during the sessions were classified according to the following scheme:

- Pointing (i.e., extending the index finger in the direction of an object, a person, or an event).
- Showing (i.e., holding up an object in the listener’s line of sight).
- Ritualised request (i.e., moving hands and arms to get a distant object).
- Conventional gestures (i.e., gestures with a culturally defined meaning and form, e.g., nodding and waving bye-bye).
- Iconic gestures (i.e., gestures that refer to objects, persons, or events, reproducing a physical or functional characteristic, e.g., flapping arms to refer to a bird).

The proportion of each category of gestures on the total number of gestures produced was computed.

### 2.4. Reliability

Independent observers assessed the intercoder reliability in 24% of the sessions (*n* = 18). We used intra-class correlation coefficients (ICC) to assess reliability. The ICC was 0.96 (with a 95% confidence interval from 0.83 to 0.99) for vocalisations, 0.98 (with a 95% confidence interval from 0.91 to 0.99) for babbling, and 0.99 (with a 95% confidence interval from 0.99 to 1) for verbal utterances. The ICC was 0.98 (with a 95% confidence interval from 0.96 to 0.99) for communicative gestures production.

### 2.5. Data Analyses

Statistical analyses were performed using IBM SPSS version 27. Since the variables were not normally distributed, we used rank transformation for the analyses. After computing the descriptives for the two groups of participants, a one-way ANOVA was used to compare children’s competence (DQ and PVB), and their spontaneous vocal and gestural production during the observation session.

We then divided the participants into two subgroups: those with a vocabulary size lower than 50 words at 24 months (late-talking group), and those with a higher vocabulary size (typically-talking group). We used a Chi-squared test to compare the proportion of late-talking toddlers in children with SCTs and TD children. Moreover, to test the hypothesis of continuity in language development, we compared the communicative production at 18 months in the late-talking and typically-talking groups. For this analysis, we considered the variables identified as predictive indices in the literature on language development: preverbal and verbal production, total gestural production, and the number of pointing gestures.

Finally, we used regression analysis to investigate which proportion of the variance in vocabulary size at 24 months was explained by children’s preverbal skills. We chose for this analysis babbling and pointing gestures, which are the variables that the literature suggests as more important to predict word onset [7]. Besides preverbal production, we considered as possible predictors early lexical abilities and DQ at 18 months.

## 3. Results

### 3.1. Comparison between Children with SCTs and TD Children at 18 Months

Children’s DQ and PVB at 18 months were significantly different. Specifically, as shown in Table 1, both DQ and PVB were significantly lower in the group of children with SCTs. Concerning their communicative production during the observation session (see Table 2), children with SCTs showed a significantly lower production of total utterances, babbling, verbal utterances, word tokens, and word types. Even gesture production was significantly lower in children with SCTs, considering the total number of communicative gestures produced and the number of pointing gestures.

**Figure 1:**
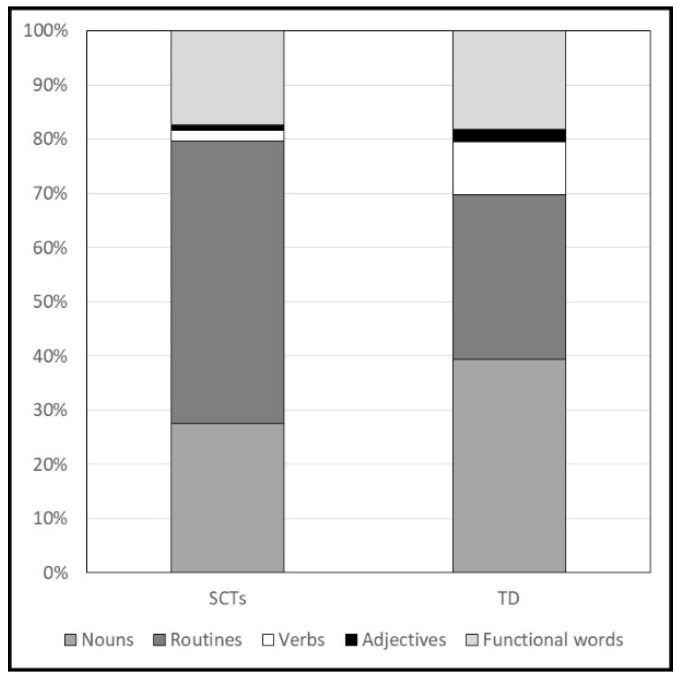
Categories of word types produced during the observation session at 18 months.

**Figure 2:**
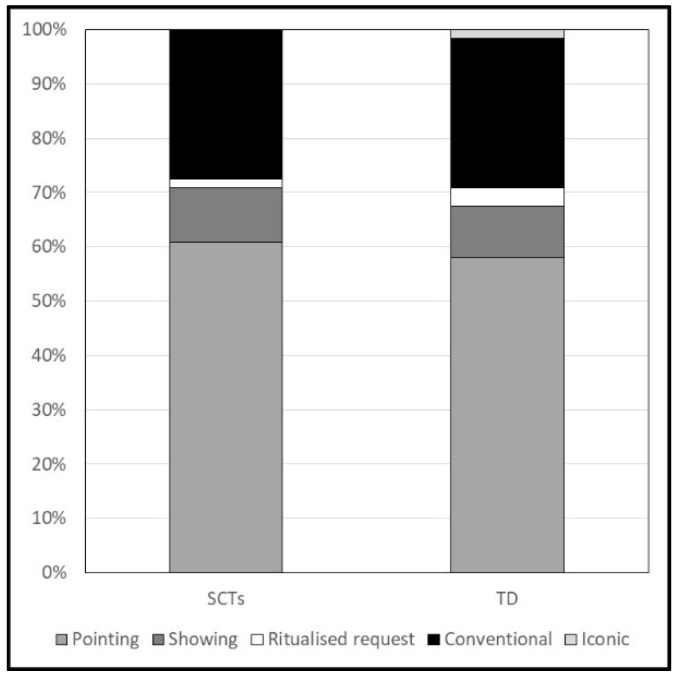
Categories of gestures produced during the observation session at 18 months.

Concerning children’s vocabulary composition, the percentage of word types uttered in each word class is reported in Figure 1 for both groups of children. The proportion of nouns (SCTs: M = 0.27; SD = 0.28. TD: M = 0.39; SD = 0.18. F = 4.22; *p* < 0.044; *η^2^* = 0.06) and verbs (SCTs: M = 0.02; SD = 0.06. TD: M = 0.10; SD = 0.10. F = 14.09; *p* < 0.001; *η^2^* = 0.19) were significantly higher in TD children. In contrast, the proportion of routines was significantly higher in children with SCTs (SCTs: M = 0.52; SD = 0.33. TD: M = 0.30; SD = 0.18. F = 11.69; *p* = 0.001; *η^2^* = 0.16). No significant differences were found in the proportion of adjectives, and function words (Fs < 1.54; ps > 0.219). Regarding the composition of gesture production in the two groups (see Figure 2), a significant difference was found only in the production of iconic gestures, which were absent in children with SCTs (SCTs: M = 0. TD: M = 0.02; SD = 0.04. F = 4.37; *p* = 0.040; *η^2^* = 0.06).

### 3.2. Language Development in Children with SCTs and TD Children at 24 Months

Children’s PVB at 24 months was significantly different between groups (F = 64.79; *p* < 0.001; *η^2^* = 0.52). In particular, the vocabulary size of children with SCTs (M = 67.23; SD = 84.39) was significantly lower than that of TD children (M = 313.25; SD = 171.89).

Children in the two groups were then divided into two subgroups: the late-talking group (i.e., toddlers with a vocabulary size lower than 50 words at 24 months), and the typically-talking group (i.e., toddlers with vocabulary size equal or higher than 50 words at 24 months). Twenty of thirty children with SCTs (67%) were late-talking toddlers, whereas only one of thirty-two TD children (3%) was a late-talking child. A Chi-squared test showed that this distribution was significantly different between groups (Chi-squared = 27.91; *p* < 0.001).

To assess the hypothesis of continuity in language development, we compared the communicative skills shown at 18 months by late-talking and typically-talking children. This analysis was performed only for the SCTs group since only one late-talking toddler was found in the TD group. Data, reported in Table 3, showed that babbling was the only measure at 18 months significantly different between late-talking and typically-talking children with SCTs. Specifically, the children in the late-talking group produced a significantly lower number of babbling utterances at 18 months.

Finally, we ran a hierarchical multiple regression analysis to test the amount of variance explained in vocabulary size at 24 months. We considered four blocks of possible predictors at 18 months: (1) babbling and pointing gestures (i.e., two preverbal measures frequently identified as predictors in the literature on language development); (2) word types (i.e., lexical skills); (3) DQ (i.e., general psychomotor development); (4) group (i.e., being a child with SCTs, or a TD child). Data showed that the independent variables selected explained 73% of the variance in PVB at 24 months (see Table 4). In particular, babbling was a significant predictor, but only after lexical skills (i.e., word types) were added to the model. In addition, DQ and group were other significant predictors of children’s vocabulary size at 24 months.

## 4. Discussion

The present study had two main aims: verifying the impact of SCTs on language development during the second year of life, and verifying the predictive role of early communicative individual differences on later linguistic outcomes in children with SCTs diagnosed before birth.

Concerning the communicative skills exhibited at 18 months, we found that the productions were significantly lower in children with SCTs than in TD children considering both vocal and gestural modalities. Toddlers with SCTs produced babbling utterances with a frequency significantly lower than TD children. This result confirms data on 8-month-old children showing a lower canonical and reduplicated babbling production in infants with SCTs [27]. As expected based on the previous literature [21], verbal production was significantly delayed in children with SCTs even at 18 months. This lexical impairment was apparent considering both data from parental reports and children’s spontaneous production in terms of word types and tokens. Vocabulary composition was the same as what was expected based on the stage of lexical development, since it generally changes with vocabulary size [40]. Nouns and routines accounted for 70% and 80% of the vocabulary in TD children and children with SCTs, respectively. According to the definition of Nelson [41], children could adopt a referential or expressive style in vocabulary development. Referential children are those for whom over 50% of their first 50 words are object names, whereas expressive children are those for whom over 50% of their first words are personal–social routines and formulas. As D’Odorico et al. [40] pointed out, more than 70% of Italian children adopt an expressive style in their first stages of vocabulary development. Therefore, the higher proportion of routines and the lower proportion of nouns and verbs in children with SCTs could be explained considering their smaller vocabulary size (a mean of 11 rather than 55 words).

The production of communicative gestures at 18 months appeared significantly lower in children with SCTs than in TD children considering both the total number of gestures and the number of pointing gestures produced. This result contrasts with previous data that found no differences in gesture production at 18 months between children with Klinefelter syndrome and TD children [33]. However, the few data available on gesture development in children with SCTs suggest a change over time in the use of gestural communicative modalities. Urbanus et al. [21] found that half of the children with SCTs between 11 and 15 months showed some difficulties in using gestures for intentional communication and imitating adult actions (as detected by parental report). In contrast, Zampini, Draghi, et al. [30] showed a higher production of pointing gestures in children with SCTs than TD children at 24 months. Therefore, the use of communicative gestures by children with SCTs seems to gradually increase with time in the passage from 12 to 24 months. We could hypothesise that in the first stages of communicative development, the production of gestures is delayed, as well as vocal production. Nevertheless, when lexical delays become more evident, and the communicative needs increase, gestures could be used by children with SCTs as a strategy to compensate for their verbal difficulties [21,33]. This compensative strategy was also found in other populations with early communicative impairments (e.g., late-talking children [6], and children with Down syndrome [42]). Concerning gesture composition, pointing accounted for nearly 60% of the gestures produced by children in both groups. The only significant difference was found in the proportion of iconic gestures, since they were absent in the production of children with SCTs. This result might be explained by hypothesising that toddlers with SCTs could show some general difficulties in expressing meanings not limited to the verbal modality. However, this point should be further investigated.

High individual variability in early communicative skills was found in both TD children and children with SCTs, as evidenced by the high standard deviations of all the measures considered. At 24 months, late-talking children (i.e., children potentially at risk for language impairments) were found in both groups, but in a significantly different proportions: only 1 child of 32 in TD children, and 20 of 30 (nearly 70%) in children with SCTs. From comparing the production at 18 months by children later identified as late-talking or typically-talking, we found a predictive index of vocabulary development. Specifically, the frequency of babbling productions was significantly higher in the children with SCTs with an adequate vocabulary size at 24 months.

A high variability characterised vocabulary size at 24 months in both groups of participants, as evidenced by standard deviations. To test which individual variables significantly contributed to explaining this variability, we investigated the contribution of babbling, pointing, early lexical skills, DQ, and group on children’s later lexical competence. As found in the literature [8], babbling appeared a significant preverbal predictor of later linguistic competence. However, this contribution lost its power when other predictors were added to the model. Specifically, early lexical skills and DQ at 18 months, in addition to group (SCTs vs. TD), were significant predictors of vocabulary size at 24 months, accounting for 73% of the variance in this measure.

### 4.1. Study Strengths and Limitations

The study’s longitudinal design is a strength of the present work. To date, only a few studies on children with SCTs allow analysing language development trajectories in this population. Another strength is the number of participants, particularly considering they were all at the same chronological age at the observation time, and they were all identified by prenatal screening, and diagnosed before birth. This ascertain method allows working on a relatively unbiased group, since participants are not children identified during investigations for developmental problems. In addition, the study of infants diagnosed before birth allows analysing their early communicative skills before any impairment in language development might emerge.

The study is not free of limitations. Owing to dropout, language outcomes at 24 months was available for 82% of children, and not for all the children participating in the 18-month session. Moreover, we considered the impact of individual factors (i.e., early communicative skills, psychomotor development, diagnosis) on children’s developmental trajectories, but we did not consider the impact of environmental variables (e.g., maternal education or socio-economic status). We choose to focus only on individual variables because introducing more independent variables in the regression model could weaken its statistical power with the number of participants involved. Future studies will consider the contribution of the environment in explaining children’s individual differences in the population of SCTs.

### 4.2. Clinical Implications

The study highlights the possibility, in children with SCTs diagnosed before birth, of identifying early those who are more at risk of developing language delays. The continuity found between early and later linguistic skills suggests that enhancing early communication abilities might support communicative intentionality, reduce frustration, and lead to better linguistic outcomes in these individuals. We also must note that not all children with STCs showed linguistic delays in the second year of life, since one-third of these toddlers were typically-talking children at 24 months. Therefore, a tailored approach based on child-specific characteristics should be adopted in working with this population.

## 5. Conclusions

The present study showed that a delay in communicative skills (both vocal and gestural) could be found as early as 18 months in children with SCTs diagnosed before birth. Moreover, we highlighted the existence of continuity between early and later linguistic skills in this population. Our results showed that toddlers with SCTs who have better communicative skills at 18 months might have better linguistic outcomes 6 months later. In addition, we showed that many individual factors might contribute to explaining lexical variability at 24 months.

## Figures and Tables

**Table 1:** DQ and PVB of children with SCTs and TD children at 18 months.

	SCTs		TD				
	*M*	*SD*	*M*	*SD*	*F*	*p*	*η^2^*
DQ ^1^	99.59	10.54	108.51	7.56	16.03	< 0.001	0.19
PVB ^2^	11.20	11.02	55.19	60.20	34.44	< 0.001	0.33

^1^ *n* = 34 for SCTs group and *n* = 35 for TD group; ^2^ *n* = 35 for SCTs group and *n* = 36 for TD group.

**Table 2:** Vocal and gestural production of children with SCTs and TD children at 18 months.

	SCTs (*n* = 38)		TD (*n* = 38)				
	*M*	*SD*	*M*	*SD*	*F*	*p*	*η^2^*
Total utterances	65.87	42.09	83.97	33.40	8.14	0.005	0.10
Vocalisations	39.63	33.67	31.82	25.15	1.82	0.182	0.02
Babbling	19.97	19.95	26.11	15.69	5.25	0.025	0.07
Verbal utterances	6.26	7.69	26.05	19.95	47.38	< 0.001	0.39
Word tokens	7.82	10.69	31.79	24.79	43.36	< 0.001	0.37
Word types	2.61	2.93	11.26	8.30	49.89	< 0.001	0.40
Total gestures	12.21	14.66	14.39	8.98	5.63	0.020	0.07
Pointing	6.74	9.42	8.05	6.97	4.11	0.046	0.05

**Table 3:** Vocal and gestural production at 18 months of children with SCTs identified as late-talking or typically-talking at 24 months.

	Late-Talking (*n* = 20)		Typically-Talking (*n* = 10)				
	*M*	*SD*	*M*	*SD*	*F*	*p*	*η^2^*
Total utterances	59.05	40.41	76.30	54.69	0.36	0.551	0.01
Vocalisations	42.10	41.25	35.20	28.37	0.10	0.757	0.00
Babbling	11.65	13.18	31.70	25.95	7.83	0.009	0.22
Verbal utterances	5.30	8.29	9.40	8.59	1.67	0.206	0.06
Word tokens	7.25	12.58	10.90	10.28	1.29	0.266	0.04
Word types	2.05	3.15	3.40	3.20	1.81	0.189	0.06
Total gestures	11.30	13.64	16.20	18.49	0.37	0.546	0.01
Pointing	7.60	10.66	6.40	7.56	0.00	0.972	0.00

**Table 4:** Predictors of vocabulary size at 24 months.

		*R^2^*	*R^2^ adj*	*F*	Δ*R^2^*	*β*
Model 1		0.26	0.24	9.65 _(2, 54)_ ***		
	Babbling					0.47 ***
	Pointing					0.19
Model 2		0.54	0.52	20.90_(3, 53)_ ***	0.28 ***	
	Babbling					0.13
	Pointing					0.10
	Word types					0.64 ***
Model 3		0.69	0.66	28.29_(4, 52)_ ***	0.14 ***	
	Babbling					0.07
	Pointing					0.04
	Word types					0.49 ***
	DQ					0.43 ***
Model 4		0.75	0.73	31.01_(5, 51)_ ***	0.07 ***	
	Babbling					0.10
	Pointing					0.04
	Word types					0.30 **
	DQ					0.34 ***
	Group (SCTs vs. TD)					− 0.34 ***

** *p* < 0.01; *** *p* < 0.001.

## Data Availability

Data will be made available on request.
